# Cancer Stem Cells of Differentiated B-Cell Malignancies: Models and Consequences

**DOI:** 10.3390/cancers3021566

**Published:** 2011-03-25

**Authors:** Emilie Gross, Anne Quillet-Mary, Loic Ysebaert, Guy Laurent, Jean-Jacques Fournie

**Affiliations:** 1 INSERM, UMR1037-Cancer Research Center of Toulouse, 31300 Toulouse, France; E-Mails: emilie_gross@hotmail.fr (E.G.); anne.quillet-mary@inserm.fr (A.Q.-M.); loic.ysebaert@inserm.fr (L.Y.); guy.laurent@inserm.fr (G.L.); 2 ERL 5294 CNRS, BP3028 CHU Purpan, 31300 Toulouse, France; 3 Université Toulouse III Paul-Sabatier, 31300 Toulouse, France; 4 Service d'Hématologie, CHU Purpan, 31300 Toulouse, France

**Keywords:** cancer stem cell, B-Lymphoma, multiple myeloma, B-CLL, differentiated B malignancy, memory B cell, reprogramming

## Abstract

The concept of cancer stem cells has revolutionized our current vision of cancer development and was validated in solid tumors and cancers of the primitive hematopoietic compartment. Proof of the principle is still lacking, however, in malignancies of differentiated B-cells. We review here the current literature, which nevertheless suggests hierarchical organizations of the tumor clone for mostly incurable B-cell cancers such as multiple myeloma, lymphomas and B-chronic lymphocytic leukemia. We propose two models accounting for cancer stem cells in these contexts: a “top-to-bottom” clonal hierarchy from memory B-cells and a “bottom-to-top” model of clonal reprogramming. Selection pressure on the growing tumor can drive such reprogramming and increase its genetic diversity.

## 1. Introduction

Pioneer work by John Dick and Dominique Bonnet has initiated a new vision of carcinogenesis by demonstrating the hierarchy of the AML malignant clone [1]. By showing that AML comprised a minor immature compartment, whose properties recapitulate those of the normal hematopoietic stem cell (HSC), they demonstrated a new model of leukemogenesis and defined the first cancer stem cell. By analogy with hierarchical organization of hematopoiesis, this concept implies that a discrete fraction of cells with stem cell features (asymmetric division) is able to indefinitely sustain the malignant progeny through self-renewing and differentiation processes. Since this initial report in the mid 90s, there has been a great enthusiasm for this concept as evidenced by the number of identified “cancer stem cells” (CSC) in varied hematological and solid tumors (chronic myelogenous leukemia (CML), acute lymphoblastic leukemia (ALL), breast cancer, brain cancer, prostate cancer, *etc.*).

Methods to identify putative cancer stem cells were initially based on defining the immature phenotype of cancer-initiating cells. The variability of cell surface phenotypes in various conditions, as well as the lack of applicability in every malignant setting, led investigators to devise novel methods of identification based on stem cell properties [2]. The side population (SP) phenotype mediated by the activity of ABCG2, an ABC transporter involved in hematopoietic stem cell biology, and detected by efflux of the Hoechst 33342 dye defines “stem cell-like cells” with drug resistant potential [3-5]. Similarly, activity of ALDH, an enzyme involved in detoxification of a variety of compounds including active metabolites of cyclophosphamide [6] and preferably expressed in primitive cell compartment [7], permits the identification of putative stem cell compartment [8,9]. Although these methods allow definition of specific primitive cell compartments, definitive proof of cancer-initiating potential is provided by serially transplanting (self-renewal) these cells and recapitulating the initial cancer heterogeneity (differentiation) in xenografted or transgenic mice models.

With the cancer stem cell concept, a new era of cancer therapy has emerged. The conventional therapies destroy differentiated cancer cells but leave intact the highly chemoresistant cancer stem cells. After eradication of the tumor progeny by therapy, the “cancer stem cells” able to switch on their mitotic division/differentiation program can drive the reemergence of the initial tumor heterogeneity [10]. Thus, full eradication of the malignant clone requires the integration of tumor heterogeneity for the design of efficient therapeutics.

The cancer stem cell concept has been fully validated in AML, ALL and some solid cancers, since the cell that received the initial oncogenic hit was a stem cell itself in such pathologies. By definition indeed, no such stem cells exist in mature lymphopathies, even if long lasting, self renewing memory cells exist. So what should be the definition of a CSC? From a clinician viewpoint it is a cell that resists all therapies and drives subsequent relapses, while a more formal definition implies a cell that is able to self-renew, migrate, differentiate and reconstitute the heterogeneity of the initial tumor. Since relapses are always part of the natural history of low grade lymphomas and myeloma, these diseases involve stem cells according to the first definition, although scientific data validating this conclusion based on the second definition are completely lacking.

**Figure 1.**
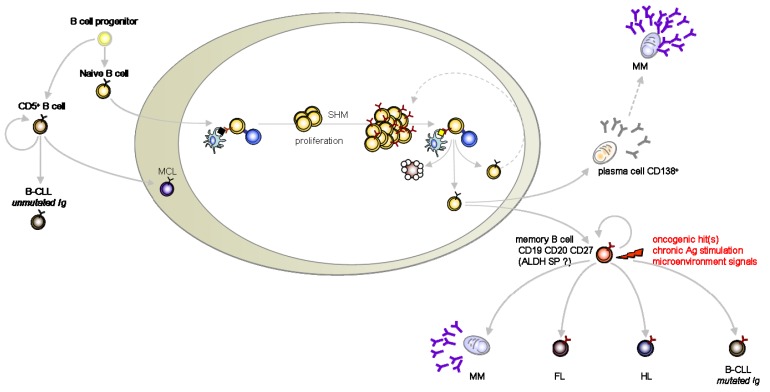
B-cell malignancies and their progenitor cells.

We chose to blunt the strict CSC definition to its more clinical version such as to take in account the evidence for cancer-initiating cells in hematopoietic B mature malignancies which are now emerging from the literature. Here we review and discuss the studies which support the CSC theory in multiple myeloma (MM), lymphomas and B-chronic lymphocytic leukemia (B-CLL) (Figure 1).

## 2. Multiple Myeloma (MM)

Along with elevated serum immunoglobulin and osteolytic bone disease, multiple myeloma (MM) is characterized by the clonal expansion and accumulation of mature and quiescent CD45^-^CD138^+^ antibody secreting neoplasic plasma cells in the bone marrow. Despite initial clinical responses induced by a wide range of cytotoxic agents, MM remains incurable with almost constant reconstitution of the malignant clone after each therapeutic round. Such a clinical scenario is usually attributed to the underlying activity of CSCs.

The hierarchy of an MM-stem cell whose progeny replenishes the MM clone was proposed a long time ago [11,12]. In these early works, malignant plasma cells derived from Balb/c mice with chronic peritoneal inflammation were engrafted in syngenic animals with a tumorigenic cell frequency as low as ∼1/1,000 cells. The self-renewal capacity of these MM-initiating cells was then formally demonstrated by their subsequent engraftment in secondary recipients. Thus, the MM population was heterogeneous, as it encompassed both self-renewing, highly proliferative stem cells and their more quiescent progeny [11-13]. A related clonogenic potential was uncovered in few MM cells from primary human MM samples [14]. First reports addressing the cancer stem cell existence in human multiple myeloma did demonstrate the transplantable engraftment of mature myeloma cells (CD45-/CD38high) in the SCID mouse implanted with human fetal bone fragments to create a humanized microenvironment (SCID-hu). Self-renewal potential of mature myeloma cells as well as recapitulation of the myeloma disease hallmarks (hypercalcemia, circulating M protein and resorption of the human bone fragment) were achieved through this approach. The myelomagenic potential of this mature compartment of the disease was further validated by the absence of engraftment with injection of plasma cells depleted blood samples [15,16].

In dichotomous studies, anti-MM-idiotype antibodies revealed the presence of a phenotypically distinct, more immature- compartment of proliferating MM B cells both blood and bone marrow from patients with MM and monoclonal gammopathy of unknown significance (MGUS, a pre-myeloma state) [17]. The Ig gene rearrangements and chromosomal abnormalities of such clonotypic MM B-cells were characterized, together with the demonstration of their ability to differentiate into plasma cells *in vitro* [18-21]. In addition, MM cells are malignant counterparts of the terminal stage (CD138^+^) of B cell lymphopoiesis. Accumulating evidence from studies with xenografted mice suggest that the MM-initiating cells are confined within a small CD19+CD138- subset differing from the CD20-CD138+ malignant bulk unable to engraft in NOD/SCID mouse model [22-24]. Further phenotypic definition of these clonotypic MM B cells delineated the myelomagenic potential to CD19+/CD138^-^/CD27+/CD20+ cells, a phenotype characteristic of memory B cells. Such CD138^-^ cells displayed not only chemoresistance but also stem cell characteristics such as ALDH^+^ and SP phenotype, constitutive Hedgehog signaling and self-renewal in serially transplanted mice [25,26]. Although a large amount of recent evidence tends to favor the B cell origin of myeloma initiating cells, discrepancies regarding the identity of myelomagenic cells, due to varying tumor transplant microenvironments (humanized *vs.* mouse), remains to be clarified in an appropriate myeloma syngenic tumor model.

Obviously, a highly specific targeting of this MM clonotypic B cell population is therefore likely to validate the CSC concept in MM, in addition to unveil new therapeutic options for this disease.

## 3. B-Lymphomas

The generic denomination of B-cell lymphoma encompasses a variety of entities (>70 in WHO classification) which pathogenesis relies on B-cell neoplasic transformation and accumulation within the lymphatic tissues. B-cell lymphomas are divided into Hodgkin lymphomas (HL) and Non-Hodgkin lymphomas (NHL), which consist of 30 different malignant entities. The most prevalent malignancies of this second group are Diffuse Large B cell lymphoma (DLBCL, 35%), Follicular Lymphoma (25%) and Mantle Cell Lymphoma (5–10%). The first formal evidence for lymphoma-initiating cells came from the detection of few transplantable lymphoma cells in mice [27]. Since then, the lymphoma stem cell hypothesis has remained largely unexplored in these diseases, although the following lines of evidence now suggest their existence in Hodgkin's, Follicular and Mantle Cell lymphomas (see below).

## 4. Hodgkin Lymphoma

Hodgkin Lymphoma (HL) is a very unique cancer in which neoplasic cells (Hodgkin Reed-Sternberg/HRS cells), comprising both multinucleated (Reed-Sternberg; RS) and single-nuclei Hodgkin cells, account for 0.1–1% of the total cells in a biopsy. These morphologically atypical tumor cells from the hematopoietic B lineage express CD30 and CD15 and lack typical sIg markers of B-cell identity. However, their B-cell origin is evidenced by the occurrence of clonal Ig heavy chain gene rearrangement and somatic mutations [28]. As for MM, HL cell lines (L428 and KM-H2) comprise a small fraction of B-cells harboring the CD20+ CD27+ memory phenotype as well as the ALDH activity involved in stemness [7]. When sorted, such CD20+ CD27+ memory B cells were able to durably generate HRS cells *in vitro* and they displayed high clonogenic and self-renewal potentials. Related clonotypic B-cells in peripheral blood samples from most HL patients were detected by light chain restriction among the CD27+ ALDHhigh cells [29]. Likewise a very recent report comparing the tumorigenic potential of multinucleated (Reed-Sternberg; RS) (M) and single nucleated cells (Hodgkin cells) (S) from two HL cell lines revealed a novel functional heterogeneity of the HL clone [28]. S cells, through their enhanced tumorigenicity in NOD/SCID mice and ability to generate both S and M cells along with low intracellular ROS concentration, high FOXO3a expression level, SP phenotype and dauxorubicine resistance, may be putative candidates for HL initiation [30,31]. Although links between mononucleated cells and clonotypic B cells from HL have to be defined, the lymphoma-initiating capacities remain to be formally proven in an appropriate murine model.

## 5. Follicular Lymphoma

The hallmark of Follicular Lymphoma (FL) is the t(14;18) translocation which causes overexpression of the anti-apoptotic Bcl-2 gene. Although this chromosomal rearrangement constitutes a founding step for FL oncogenesis, additional molecular and cellular events are required for full blown malignant transformation. FL remains an incurable disease, with frequent relapses replenishing the initial tumor bulk after chemotherapies. As for HL, very few studies investigated a potential hierarchy in the FL clone.

The first element concerning the cellular origin of FL is the presence of a pre-malignant cell subset in peripheral blood from healthy adults. This subset is composed of oligoclonal and long-lived normal B-cells which harbor the t(14;18) translocation and a CD27^+^IgD^+^ phenotype. Together with some unusual features of memory B cells (such as allelic paradox), these characteristics are typical of the FL genome and phenotype [32]. Little is known on the genesis of such premalignant clones, except for that they progressively expand in farmers exposed to pesticides epidemiologically linked to NHL [33]. Investigations of this atypical “memory-like” and pre-malignant FL seed in murine models are nevertheless required to validate FL stemness.

The second element suggesting a FL hierarchy is the persistence of a t(14;18)^+^ cell subset in lymph nodes from most FL patients in remission [34]. In line with the clinical course of FL, these remission-relapse episodes might thus reflect post-therapeutic replenishment of the malignant clone. Whether the t(14;18)^+^ cell persistence is mediated by intrinsic chemoresistance (due to stemness features such as ALDH^+^ and SP^+^) or by environment-mediated drug resistance remains controversial. In favor of the intrinsic chemoresistance, however, preliminary data revealed that some FL cell lines and biopsies displayed an ABCG2^+^ SP^+^ subset that was highly chemoresistant to gemcitabine [35].

In conclusion, the hierarchy concept is still in its infancy for FL, and much scientific evidence is still lacking to formally validate this hypothesis. They comprise, *inter alia*, the characterization of the stemness signature of the pre-FL cell population and the identification of second hits leading to FL pathogenesis.

## 6. Mantle Cell Lymphoma

Mantle Cell Lymphoma (MCL) is characterized by the expansion of neoplastic CD5+, cyclin D1+ (due to a t(11;14) translocation) cells which accumulate in the lymph nodes, bone marrow, spleen, gastrointestinal tract and to a lesser extent in blood. The sole report suggesting a hierarchical organization of the MCL clone described SP cells in (IL14α × c-Myc) double transgenic mice, which developed MCL. The surprisingly large MCL SP compartment in this model exhibited both cytogenetic hallmarks and stemness features of MCL including longer telomeres, higher *in vitro* clonogenic and self-renewal potential as well as increased MCL-initiating capacities in xenografted mice compared to non-SP cells [36]. To fully extrapolate this concept to the pathogenesis of human MCL however, this SP hierarchical organization of the MCL clone remains to be validated in MCL biopsies.

## 7. Chronic Lymphocytic Leukemia

B-cell Chronic Lymphocytic Leukemia (B-CLL) is characterized by the clonal expansion and accumulation of CD19^+^ CD5^+^ malignant -though quiescent- lymphocytes in the blood, the bone marrow and the lymphoid tissues. Accumulating evidence suggests that the proliferative compartment of this malignancy resides in specialized structures from lymphoid tissues called proliferation centers [37,38]. This unique feature of B-CLL reflects its strong dependence to micro-environmental signals and precludes most classic mice models. Among the various factors involved in B-CLL prognosis, the mutational status of BCR defines two types of disease: B-CLL with mutated (associated with a good prognosis) or with unmutated Ig (associated with a poor prognosis) [39]. Somatic hypermutations of immunoglobulins occurs during the germinal center (GC) reaction. The mutational, phenotypic and transcriptomic profiles displayed by mutated IgVH B-CLL support the leading hypothesis that these cells may arise from the leukemic transformation of IgM+IgG+CD27+ memory B cells. The identity of the normal B counterpart of unmutated IgVH B-CLL is less clear. Whereas transcriptomic analysis tends to relate them to post-GC memory cells, unmutated IgVH B-CLL cells were shown to share many similarities with the murine B1 cells residing in the marginal zone of which the activated profile is GC-reaction independent (see [38] for review). Further confirmed by the failure of previous studies to evidence the involvement of HSC in B-CLL [40, 41], these main speculations regarding the identity of a differentiated normal precursor of B-CLL may delineate a previously unexpected hierarchical organization of the leukemic clone [42].

In line with the CSC concept in B-CLL, this cancer remains incurable due to post-therapeutic re-emergence of the leukemic cell clone. As for MM and FL, this clinical evolution could result from a “B-CLL stem cell” clone with high drug-resistant and post-therapeutic replenishing potentials. This intraclonal heterogeneity of the B-CLL clone was tracked by monitoring its SP phenotype. Others and we have demonstrated the existence of few drug-resistant CD19^+^CD5^+^ SP cells in virtually all patients [43,44]. Their identical cytogenetic abnormalities indicated that such SP were clonally related to the rest of the B-CLL cells. Furthermore, the post-therapeutic amplification of these SP suggested their implication in the relapse. Of note, however, the chemotherapeutic pressure selected an evolutive conversion of some non-SP cells into SP cells over-expressing both ABCG2 and the stem cell marker BMI-1 [45].

These recent studies of the B-CLL disease uncovered a hitherto unattended heterogeneity of the leukemic clone regarding stem cell-like features such as the SP phenotype, multi drug resistance and BMI-1 over-expression. Whether this heterogeneity is hierarchical remains to be determined, for example through investigation of the B-CLL-initiating potential of its SP fraction in murine model. Engraftment of primary B-CLL cells, being laborious in a xenograft model, an alternative approach for exploring functional heterogeneity of the tumorigenic potential of SP cells may involve syngenic transplantation from transgenic mice recapitulating the B-CLL disease (Eμ-TCL1 or the MDR-/-) [46,47].

## 8. Memory B Cells: the Usual Suspects

In search for cancer-initiating cells, memory B-cells are usual suspects for the pathogenesis of differentiated B-cell malignancies (MM, HL, FL, mutated B-CLL) [32,38,48] (Figure 1). Likewise, unmutated B CLL cells as well as MCL are orthopically related to long-lived and self-replenishing CD5^+^ murine cells [48]. Although self-renewal is confined to the primitive stem cell compartment in most tissues, adaptive immunity maintains life-long protection through self-renewal of memory B and T lymphocytes [49]. Supporting this parallel, memory T cells can undergo asymmetric division [50] and self-renewal programs represent a gene signature shared by memory B and T lymphocytes as well as by HSC [51]. Of note, BMI-1 over-expression is also shared by both memory B cells and HSC [51].

Here, we propose that memory B cells represent a cancer stem cell compartment in most mature B malignancies. These cells represent an expanded and self-renewing reservoir refueling tumor growth through a hierarchical paradigm matching the HSC concept in AML and CML. Transformation of the B-cell memory compartment by chromosomal translocation (for FL) and additional events (e.g. microenvironmental or chronic antigenic stimuli) [33,38] might drive the generation of malignant progenies. In such a paradigm, the type of transforming molecular event targeting the same memory B cell might contribute to determine the various malignant entities produced (FL, CLL, HL, MM). Whether this “transformed” memory B cell possesses a drug-resistant phenotype remains to be demonstrated. We speculate that the stemness of memory cells involves their drug-resistance transcriptional programs.

## 9. Molecular Reprogramming

Although consistent pieces of evidence tend to make memory B-cells good candidates for mature B CSC, accumulating data demonstrate the impressive plasticity of B-cell lineage differentiation. Illustrations of such reprogramming events in cancer are provided by AML and CML pathogenesis, where committed progenitors can acquire stem cell properties [52,53]. Although the natural occurrence of such B-cell differentiation reprogramming has never been documented, the bioactivity of reprogramming transgenes has formally demonstrated the extraordinary plasticity of lymphocytes. Differentiated B cells were reprogrammed into macrophages upon introduction of CEBPα/β combined to extinguished expression of Pax5, a B-cell identity gene [54]. Furthermore, conditional mutant Pax5 gene triggered the retro-differentiation of mature B-cell into uncommitted lymphoid progenitors in mice. In addition to the development of immature and aggressive lymphoma, it also triggered an efficient B-to-T cell conversion when transplanted in mice lacking lymphoid cells [55]. Along studies of induced pluripotent stem cells (iPS), terminally differentiated B cells were reprogrammed to pluripotency through ectopic expression of Oct4, Sox2, Klf4 and c-MYC in conjunction with interruption of B-specific transcriptional program by specific knockdown of *Pax5* or exogenous expression of CEBPα/β [56].

Although the occurrence of reprogramming phenomena in mature B cell lymphoma remains unknown, specific gene expression may confer stemness properties. This might occur during malignant progression or initiation, enabling the fraction of cancer-initiating cell to gain, independently of their inherent differentiation, self-renewal and replenishing activities. For instance, Bmi-1 over-expression could represent such a reprogramming event in B-CLL. Supporting this hypothesis, mimicking BMI-1 activity by deleting its target genes in multi-potent progenitors allowed these cells to acquire a longterm hematopoietic repopulating ability that was otherwise the HSC compartment's privilege [57]. In addition, the c-Myc oncogene, which is frequently deregulated in B-lymphoma, is also involved in controlling the balance between self-renewal and differentiation in HSC [58]. As a plausible proof of concept for the function of c-Myc in lymphoma stem cells, Eμ-MYC lymphoma cells were almost homogenously able to initiate lymphomas in transplantation experiments [59]. It is thus possible that c-Myc was a reprogramming factor which conferred stemness to the malignant cells in these mice.

## 10. Conclusion

The CSC concept established in AML has yielded a new understanding of carcinogenesis and relapses. The underlying mechanism was based on a hierarchical structure of the malignant clone from this highly primitive compartment of hematopoiesis. With malignancies of the differentiated B lymphocytes, however, this concept still remains to be validated, although the lines of evidence reviewed here do support its validity. These studies recurrently point to the putative involvement of memory B cells as CSC in differentiated B-cell malignancies as diverse as NHL, MM, HL and CLL. Although in these diseases, the hierarchical model implies that CSC generates tumor heterogeneity through the developmental plasticity of memory B-cells, other models of tumor clone organization can also be proposed.

**Figure 2.**
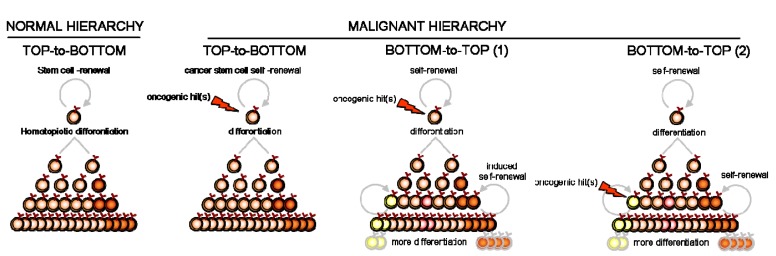
Models of clonal progenies in normal and malignant contexts. Note that the difference between bottom-to-top models 1 and 2 is the target of the oncogenic hit.

We infer that cellular reprogramming can disrupt the initial clone pyramid and initiate the tumor in a reverse, “bottom-to-top” fashion. This does not necessarily refute the hierarchical paradigm of CSC, but rather requires a secondary, inducible “bottom-to-top” reprogramming of new clonal hierarchies. Although such “bottom-to-top” reprogramming has already been demonstrated for committed progenitors of AML and CML [52,53,60], evidence for the natural occurrence of molecular reprogramming in the pathogenesis of B-differentiated malignancy is still lacking. The fludarabine-induced switch of some non-SP cells into SP cells in B-CLL [43] may delineate such a “reprogramming” plasticity in malignant mature B cells. This induced reprogramming might not be restricted to responses to chemotherapeutic exposure [61,62] however, since different selective pressures usually edit oncogenesis and cancer progression. Oncogene insult [63,64] as well as hypoxia [65,66], microenvironmental signal [67,68] or cell migration [69,70] (e.g., epithelial-mesenchymal transition) might also induce “bottom-to-top” reprogramming. Since most diagnosed cancers have already been exposed to these pressures for growth, we hypothesize that reprogramming within the malignant clone is most likely to have occurred and yielded a heterogeneous and diversified cell progeny. However, classic (top-to-bottom) hierarchical reprogramming from the same upstream CSC is less prone to diversify this organization than the reverse (bottom-to-top) reprogramming from daughter cells (Figure 2).

Although recent reports support this concept [59], future studies from our and other laboratories will explore the pressure-induced reprogramming of highly differentiated cells into cancer-initiating cells within the context of mature B-cell lymphomas.
